# Association of glycosylated haemoglobin HbA1c levels with outcome in patients with COVID‐19: A Retrospective Study

**DOI:** 10.1111/jcmm.16431

**Published:** 2021-03-10

**Authors:** Shuai Yuan, Huaping Li, Chen Chen, Feng Wang, Dao Wen Wang

**Affiliations:** ^1^ Division of Cardiology Department of Internal Medicine Tongji Hospital Tongji Medical College Huazhong University of Science and Technology Wuhan China; ^2^ Hubei Key Laboratory of Genetics and Molecular Mechanisms of Cardiological Disorders Wuhan China

**Keywords:** COVID‐19, diabetes, HbAlc, mortality

## Abstract

Patients with hyperglycemia tend to be susceptible to Coronavirus disease 2019 (COVID‐19). However, the association of HbA1c level with outcome of COVID‐19 patients was unclear. We performed a retrospective study of 2880 cases of COVID‐19 admitted in Tongji Hospital, Wuhan, China, among which 922 had detected the HbA1c levels. We found that COVID‐19 patients with either lower levels of HbAlc (3%‐4.9%) or higher levels of HbAlc (≥6%) were associated with elevated all‐cause mortality. Meanwhile, we observed that HbAlc levels were highly correlated with haemoglobin (Hb) and total cholesterol (TC) (*P* < .0001), moderately correlated with albumin (ALB) and high‐sensitive C reaction protein (hs‐CRP) (0.0001 < *P*<.001), and relatively low correlated with low‐density lipoprotein cholesterol (LDL‐C) (.001 < *P*<.01). These associated cofactors might together contribute to the clinical outcome of COVID‐19 patients. Furthermore, the mortality was higher in COVID‐19 patients with newly diagnosed diabetes mellitus (DM) compared with COVID‐19 patients with history of DM. Moreover, in patients with history of DM, the mortality was decreased in patients treated with anti‐hyperglycaemic drugs. In summary, our data showed that the in‐hospital mortality was increased in COVID‐19 patients with lower or higher levels of HbAlc. Meanwhile, initiation of appropriate anti‐hyperglycaemic treatment might improve the clinical outcome in COVID‐19 patients.

## 1 INTRODUCTION

The coronavirus disease 2019 (COVID‐19) is now rapidly spreading throughout the world.^1^ According to the published data, older patients with underlying illness such as diabetes and cardiovascular diseases tend to be susceptible to COVID‐19 and become severely ill.^2^, ^3^, ^4^ Specifically, a recent study found that subjects with type 2 diabetes required more medical interventions and had a significantly higher mortality and multiple organ injury than the non‐diabetic individuals. Further, the study also found that well‐controlled blood glucose (BG, glycaemic variability within 3.9 to 10.0 mmol/L) was associated with markedly lower mortality compared to individuals with poorly controlled BG (glycaemic variability exceeding 10.0 mmol/L) during hospitalization.^5^ These findings provide clinical evidence for the concept that improved glycaemic control with better outcome in patients with COVID‐19 and pre‐existing type 2 diabetes.

While the close association between diabetes and increased mortality becomes clear, glycaemic‐control achievements evaluation using glucose levels (FBG fasting glucose) are sometimes problematic. Because blood glucose levels are highly variable and easily effected by short‐term stress such as infection, glucocorticoid therapy and Somogyi phenomenon. Notably, the Somogyi effect is the tendency of the body to react to extremely low blood sugar (hypoglycaemia) by overcompensating, resulting in high blood glucose levels.^6^, ^7^ Therefore, FBG level alone might be unable to efficiently reflect the true glycaemic metabolism in COVID‐19 patients. On the other hand, strict blood glucose surveillance or OGTT test might accelerate the shortage of medical resources during the COVID‐19 pandemic.

HbA1c is produced by a non‐enzymatic reaction that occurs between glucose and haemoglobin.^8^ As plasma glucose increases, the fraction of HbA1c increases in a predictable way. This serves as a surrogate marker for average blood glucose levels over the previous 3 months prior to the measurement.^9^ In 2009, the American Diabetes Association included HbA1c ≥ 6.5% (48 mmol/mol) as a diagnostic criterion for diabetes.^10^, ^11^, ^12^ Fasting is not needed for HbA1c assessment and no acute perturbations (eg stress, diet and exercise) affect HbA1c.^13^ Moreover, HbA1c captures chronic hyperglycaemia better than two assessments of fasting or 2‐h oral glucose tolerance test plasma glucose.^13^ However, to date, limited information is available regarding HbAlc levels and clinical outcome of COVID‐19, which might be due to problems in standardization and variations in styles of HbA1c test among multiple‐centred studies.

In this report, we performed a retrospective longitudinal study from a cohort of 992 confirmed COVID‐19 cases enrolled in single‐centred Tongji hospital in Wuhan, China focusing on the association between plasma HbAlc level and clinic outcome in COVID‐19 patients. In addition, by using the cut‐off value of HbA1c ≥ 6.5%, we sought to investigate the mortality of COVID‐19 patients with in‐hospital newly identified DM in comparison with previously diagnosed DM. Moreover, we further assessed the mortality of COVID‐19 DM patients treated with different anti‐hyperglycaemic drugs.

## 2 METHODS

### 2.1 Ethics

The study, approved by the Ethics Review Board of Tongji Hospital and Tongji Medical College, conforms to the principles outlined in the Declaration of Helsinki. Written and informed consent forms were waived by the ethics boards of the hospitals.

### 2.2 Study design

The study included patients with COVID‐19 diagnosed between 10 January 2020 and 30 March 2020. COVID‐19 was diagnosed based on chest computed tomography (CT) manifestations and/or reverse transcription‐polymerase chain reaction (RT‐PCR) following the criteria of the New Coronavirus Pneumonia Prevention and Control Program (5th edition) published by the National Health Commission of China and WHO interim guidance. A total of 2880 patients with COVID‐19 were initially screened for the study, and 922 of them had HbA1c level detected.

### 2.3 Data collection

All clinical data (including basic information, clinical manifestations, laboratory findings, treatments and outcome during hospitalization) were obtained from patients’ electronic medical records. The laboratory findings included routine blood test, fasting blood glucose (FBG) and HbA1c, C‐reactive protein (CRP), D‐dimer for liver function, kidney function, coagulation function and inflammation analysis.

### 2.4 Statistical analysis

All statistical analysis was performed using SPSS 21.0 for Windows (SPSS Inc, Chicago, IL, USA). Categorical variables were presented as number (percentage), and continuous variables were presented as median (interquartile range). Categorical variables were compared using the chi‐squared test or Fisher exact test. Normally and abnormally distributed continuous variables were compared using the Student's *t* test and the Mann‐Whitney *U* test, respectively. Kruskal‐Wallis ANOVA tests (nonparametric unpaired) was used among multiple groups. To assess the significance of the correlations, Spearman rank correlation coefficient was calculated. Unless otherwise stated, a value of *P* (or corrected P in case of multiple groups) <.05 was considered statistically significant.

## 3 RESULTS

### 3.1 Association of HbAlc levels with mortality of COVID‐19 patients

**FIGURE 1:**
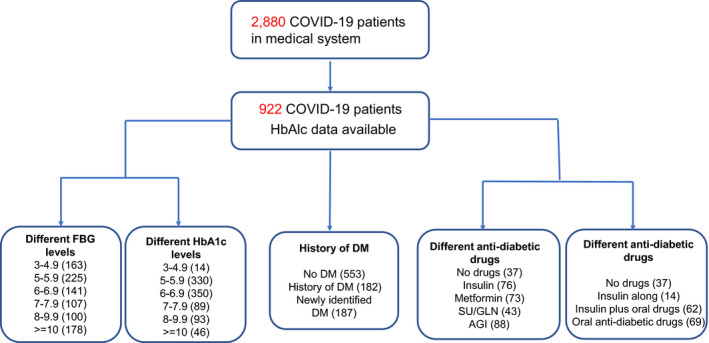
Study design. A schematic overview illustrating participant enrolment in the study

**FIGURE 2:**
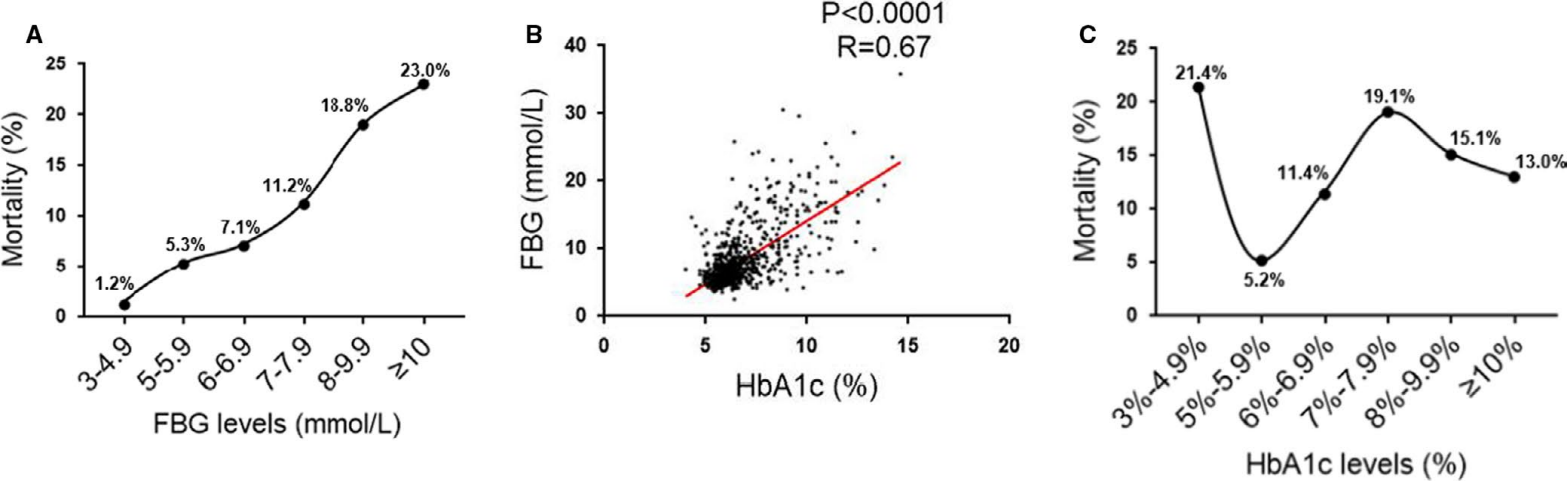
Association of HbAlc level with mortality of COVID‐19 patients. A, All‐cause mortality in patients with different FBG levels. B, Correlation analysis between FBG and HbAlc levels. C, All‐cause mortality in patients with different HbAlc levels

A total of 2880 confirmed COVID‐19 patients were admitted to Tongji hospital, Wuhan, China from 10 January 2020 to 30 March 2020 during the pandemic, among which 922 patients had HbAlc level examined (Figure 1). We first analysed mortality by dividing these 922 COVID‐19 patients into 6 subgroups based on FBG levels (Table 1). We found that higher levels of FBG expression were associated with increased COVID‐19 mortality (Figure 2A) while lower FBG (3‐4.9 mmol/L) was associated with the best clinical outcome (with mortality of 1.2%). We then analysed mortality by dividing these 922 COVID‐19 patients into 6 subgroups based on HbAlc levels (Table 2). Unexpected, though HbAlc levels and FBG levels were highly correlated (Figure 2B), lower levels (3%‐4.9%) of HbAlc expression was associated with increased mortality (21.4%). HbAlc levels between 5%‐5.9% were associated with relatively improved outcome of patients with COVID‐19 while higher HbAlc levels (≥6%) were associated with increased all‐cause mortality (Table 2, Figure 2C). Furthermore, logistic regression analysis was performed and the results showed that age (OR = 1.050, [1.030‐1.071]), gender (OR = 2.345, [1.480‐3.717]), HbA1c (OR = 1.171 [1.026‐1.337]) and coronary heart disease (OR = 2.288, [1.235‐4.240]) had effects on mortality, while other comorbidities had no effect on mortality.

**TABLE 1:** The characteristics and clinical outcome of COVID‐19 patients with different FBG levels

FBG levels (mmol/L)	3‐4.9 (n = 163)	5‐5.9 (n = 225)	6‐6.9 (n = 141)	7‐7.9 (n = 107)	8‐9.9 (n = 100)	>=10 (n = 178)	*P* value
Demographics and clinical characteristics							
Age (y)	58 (43‐67)*	64 (51‐70)	65 (52‐70)	64 (53‐73)	63 (56‐70)	66 (57‐73)	<.001
Female (%)	82 (50.3)	126 (56)	68 (48.2)	57 (53.3)	41 (40.6)	84 (47.2)	.155
Comorbidity							
Hypertension (%)	39 (23.9)	69 (30.7)	40 (28.4)	34 (31.8)	46 (45.5)*	69 (38.8)*	.003
Coronary artery disease (%)	11 (6.7)	18 (8.0)	15 (10.6)	5 (4.7)	12 (11.9)	7 (3.9)	.088
COPD (%)	4 (2.5)	2 (0.9)	1 (0.7)	1 (0.9)	1 (1.0)	2 (1.1)	.827
Malignancy (%)	4 (2.5)	2 (0.9)	2 (1.4)	2 (1.9)	3 (3)	4 (2.2)	.727
Chronic kidney disease (%)	3 (1.8)	4 (1.8)	1 (0.7)	1 (0.9)	0 (0)	0 (0)	.348
Cerebrovascular disease (%)	3 (1.8)	14 (6.2)	6 (4.3)	3 (2.8)	8 (7.9)	6 (3.4)	.143
Temperature (°C)	36.5 (36.2‐36.8)	36.6 (36.3‐37.0)	36.7 (36.3‐37.5)	36.7 (36.4‐37.3)	36.6 (36.3‐37.0)	36.6 (36.2‐37.2)	.015
Respiratory (/min)	20 (20‐21)	20 (20‐22)	20 (20‐22)	20 (20‐24)	21 (20‐25)	20 (20‐25)	<.001
Pulse (/min)	90 (80‐98)	90 (80‐102)	92 (80‐105)	90 (80‐100)	90 (80‐108)	90 (80‐106)	.397
Diastolic blood pressure (mmHg)	82 (73‐94)	79 (72‐89)	80 (72‐90)	77 (70‐85)	80 (70‐90)	80 (70‐89)	.058
Systolic blood pressure (mmHg)	128 (117‐145)	126 (116‐144)	131 (117‐146)	129 (120‐140)	127 (119‐141)	135 (120‐147)	.264
Body mass index (BMI)	23.1 (20.8‐25.4)	23.7 (21.1‐25.4)	24.5 (23.0‐26.4)	23.4 (21.5‐25.4)	23.8 (22.0‐25.1)	24.4 (21.7‐26.4)	.048
Laboratory findings							
White blood cell (*10^9/L)	5.7 (4.6‐6.9)	5.7 (4.3‐7.7)	5.6 (4.5‐7.9)	6.5 (4.6‐7.9)	6.5 (4.7‐8.6)	6.9 (5.2‐9.5)*	<.001
Red blood cell*10^12/L)	4.1 (3.7‐4.5)	4.1 (3.7‐4.5)	4.2 (3.8‐4.6)	4.2 (3.7‐4.6)	4.1 (3.7‐4.6)	4.1 (3.6‐4.5)	.674
Neutrophil (*10^9/L)	3.4 (2.6‐4.5)	3.7(2.6‐5.3)	4.1 (2.7‐5.7)	4.6 (2.9‐6.4)	4.7 (3.3‐7.1)*	5.2 (3.5‐8.3)*	<.001
Haemoglobin (g/L)	126 (117‐137)	123 (112‐135)	125 (115‐138)	128 (117‐140)	126 (117‐141)	127 (114‐139)	.348
Platelet (*10^9/L)	226 (173‐290)	229 (178‐275)	205 (157‐259)	208 (154‐295)	203 (166‐253)	213 (154‐280)	.068
Alanine transaminase (U/L)	20.0 (13.0‐37.0)	20.0 (13.0‐31.5)	23.0 (14.0‐35.0)	23.0 (15.0‐41.0)	29.0 (16.0‐44.5)*	24.0 (16.0‐37.0)	.003
Aspartate transaminase (U/L)	23.0 (18.0‐30.0)	22.0 (17.0‐34.0)	25.0 (19.0‐39.5)	27.0 (20.0‐37.0)	31.0 (20.0‐45.5)*	25.0 (18.0‐38.3)	<.001
Total bilirubin (umol/L)	8.1 (6.1‐10.7)	8.7 (6.1‐12.5)	8.5 (6.2‐11.9)	8.4 (6.5‐11.7)	10.6 (6.6‐14.3)	9.4 (6.8‐14.3)	.011
Albumin (g/L)	39.1 (34.4‐42.2)	37.4 (33.0‐40.7)	36.7 (33.8‐39.9)	34.2 (30.8‐39.8)	34.5 (31.3‐38.2)*	32.5 (29.5‐36.3)*	<.001
Globulin (g/L)	30.8 (27.6‐33.9)	31.0 (28.2‐34.3)	32.3 (29.6‐35.8)	33.4 (29.2‐36.9)*	33.7 (30.5‐36.7)*	33.4 (30.1‐37.9)*	<.001
Creatinine (mmol/L)	69.0 (56.0‐81.0)	66.0 (55.0‐81.0)	68.0(55.0‐81.5)	70.0(55.0‐86.0)	70.0 (59.0‐86.0)	67.0 (54.0‐91.3)	.735
Blood urea nitrogen (mmol/L)	4.3 (3.5‐5.3)	4.3 (3.2‐5.7)	4.2 (3.2‐6.3)	4.5 (3.4‐6.1)	4.9 (3.7‐6.4)	5.9 (4.3‐8.7)*	<.001
Uric acid (umol/L)	279.7 (238.0‐342.3)	258.0 (206.1‐323.0)	253.0 (198.5‐332.5)	244.0 (173.4‐340.2)	243.0 (187.7‐324.3)	234.5 (165.9‐296.7)	<.001
Total cholesterol (mmol/L)	4.1 (3.4‐4.8)	3.9 (3.2‐4.5)	3.7 (3.2‐4.3)	3.6 (3.1‐4.4)	3.7 (3.1‐4.3)	3.8 (3.1‐4.5)	.002
Total triglycerides (mmol/L)	1.1 (0.9‐1.7)	1.2 (1.0‐1.8)	1.2 (0.9‐1.7)	1.3 (1.0‐2.0)	1.5 (1.1‐2.4)	1.7 (1.2‐2.2)*	<.001
High‐density lipoprotein cholesterol (mmol/L)	1.1 (0.9‐1.3)	1.0 (0.9‐1.2)	0.9 (0.8‐1.1)	0.9 (0.8‐1.0)*	0.9 (0.7‐1.1)*	0.8 (0.7‐1.0)*	<.001
Low‐density lipoprotein cholesterol (mmol/L)	2.7 (2.0‐3.3)	2.5 (2.0‐3.0)	2.4 (1.9‐2.9)	2.4 (1.9‐2.8)	2.2 (1.6‐2.8)	2.4 (1.7‐3.2)	.003
K+ (mmol/L)	4.3 (4.0‐4.6)*	4.2 (3.9‐4.4)	4.2 (3.9‐4.5)	4.1 (3.7‐4.5)	4.0 (3.7‐4.4)	4.3 (3.9‐4.7)	<.001
Lactate dehydrogenase (U/L)	210.0 (178.0‐256.0)	231.0 (187.0‐316.5)	250.0 (200.5‐361.5)	280.0 (219.0‐328.0)*	302.0 (215.5‐441.5)*	303.0 (232.3‐442.5)*	<.001
Prothrombin time (s)	13.5 (13.0‐13.9) *	13.7 (13.2‐14.3)	13.8 (13.3‐14.5)	14.0 (13.5‐14.7)	13.8 (13.3‐14.9)	14.3 (13.5‐15.3)*	<.001
Activated partial thromboplastin time (s)	38.7 (36.1‐41.7)	38.9 (35.8‐43.4)	39.9 (36.6‐43.3)	39.1 (36.8‐43.0)	38.5 (36.5‐43.0)	38.4 (34.7‐42.6)	.210
D‐Dimer (ug/ml)	0.5 (0.3‐1.2)	0.6 (0.3‐1.6)	0.8 (0.4‐1.8)	0.7 (0.4‐1.9)	1.0 (0.5‐2.5)*	1.4 (0.6‐2.9)*	<.001
Interleukin 6 (pg/ml)	5.6 (2.8‐15.1)	6.6 (3.0‐32.0)	13.0 (4.5‐45.0)	7.7 (2.6‐31.9)	17.0 (5.6‐50.0)	13.3 (4.7‐42.5)	<.001
Interleukin 8 (pg/ml)	10.8 (7.5‐21.2)	12.6 (8.1‐22.5)	17.1 (10.1‐28.2)	15.1 (8.0‐25.9)	16.7 (9.3‐29.5)	15.6 (9.3‐37.7)	.002
Tumour necrosis factor‐α (pg/ml)	8.2 (6.4‐10.8)	7.8 (6.1‐9.8)	8.8 (6.4‐11.9)	8.3 (6.1‐10.8)	8.8 (7.1‐12.2)	9.0 (6.8‐12.3)*	.024
Interleukin‐1β (pg/ml)	8.6 (6.3‐11.3)	8.6 (6.7‐11.3)	8.9 (6.3‐13.9)	7.0 (5.7‐13.2)	7.8 (6.3‐11.4)	8.3 (6.9‐15.3)	.875
High‐sensitive C reaction protein (pg/ml)	2.5 (0.8‐14.8)*	9.5 (1.7‐40.8)	26.1 (4.2‐69.7)*	22.5 (2.9‐62.7)	36.8 (8.1‐106.6)*	44.4 (6.1‐97.5)*	<.001
Erythrocyte sedimentation rate (mm/h)	16.0 (7.0‐35.0)	24.0 (9.8‐43.0)	32.0 (13.3‐60.8)	28.0 (13.8‐55.3)	37.5 (16.3‐68.3)	34.5 (22.0‐70.8) *	<.001
Myoglobin (ug/L)	34.1 (25.3‐51.8)	36.8 (27.7‐57.9)	41.3 (28.4‐73.9)	45.9 (26.9‐91.2)	50.2 (28.6‐133.3)	54.3 (27.7‐129.1)*	<.001
Creatine kinase (U/L)	66.0 (43.5‐90.0)	64.0 (41.0‐99.0)	70.0 (41.0‐129.0)	61.0 (43.0‐97.0)	72.5 (43.5‐195.0)	63.5 (34.0‐138.3)	.518
Creatine kinase‐MB (U/L)	0.6 (0.4‐1.0)	0.7 (0.5‐1.2)	0.7 (0.4‐1.3)	0.8 (0.4‐1.3)	0.8 (0.4‐1.7)	0.8 (0.5‐1.5)	.077
HbA1c (%)	5.7 (5.5‐6.0)*	6.0 (5.7‐6.2)	6.1 (5.8‐6.5)	6.3 (5.9‐6.7)*	6.7 (6.2‐7.6)*	8.3 (6.6‐9.6)*	<.001
Treatments							
Metformin (%)	5 (3.1)	11 (4.9)	14 (9.9)	8 (7.5)	16 (15.8)*	58 (32.6)*	<.001
Insulin (%)	5 (3.1)	5 (2.2)	8 (5.7)	7 (6.5) *	14 (13.9)*	67 (37.6)*	<.001
SU/GLN (%)	7 (4.3)	4 (1.8)	5 (3.5)	3 (2.8)	5 (5.0)	31 (17.4)*	<.001
A‐GI (%)	5 (3.1)	15 (6.7)	19 (13.5) *	10 (9.3)	26 (25.7)*	74 (41.6)*	<.001
Pioglitazone (%)	0 (0)	1 (0.4)	4 (2.8)	0 (0)	2 (2.0)	4 (2.2)	.052
DPP‐4I (%)	0 (0)	3 (1.3)	3 (2.1)	1 (0.9)	3 (3.0)*	11 (6.2)*	.004
SGLT‐2I (%)	0 (0)	1 (0.4)	0 (0)	0 (0)	0 (0)	2 (1.1)	.611
Glucocorticoid (%)	28 (17.2)*	76 (33.8)	63 (44.7)*	46 (43.0)	53 (52.5)*	91 (51.1)*	<.001
Oxygen therapy (%)	110 (67.5)	150 (66.7)	105 (74.5)	86 (80.4)*	75 (74.3)	146 (82)*	.003
Ventilator (%)	4 (2.5)*	19 (8.4)	23 (16.3)*	20 (18.7)*	29 (28.7)*	50 (28.1)*	<.001
Intubate (%)	2 (1.2)	4 (1.8)	7 (5.0)	6 (5.6)	8 (7.9)*	26 (14.6)*	<.001
Mortality (%)	2 (1.2)*	12 (5.3)	10 (7.1)	12 (11.2)	19 (18.8)*	41 (23)*	<.001

Abbreviations: A‐GI, Alpha‐glycosidase inhibitors; COPD, chronic obstructive pulmonary disease; DPP‐4I, Dipeptidyl peptidase‐4 inhibitors; GLN/SU, Sulphonylureas/glinides; K+, potassium; SGLT‐2I, Sodium‐glucose cotransporter‐2 inhibitors.

* *P* < .05 vs FBG levels among 5‐5.9 mmol/L.

**TABLE 2:** The characteristics and clinical outcome of COVID‐19 patients with different HbA1c levels

HbA1c levels	3%‐4.9% (n = 14)	5%‐5.9% (n = 330)	6%‐6.9% (n = 350)	7%‐7.9% (n = 89)	8%‐9.9% (n = 93)	>=10% (n = 46)	*P* value
Demographics and clinical characteristics							
Age (y)	58 (48‐72)	56 (43‐66)	66 (58‐73)*	66 (57‐71)*	66 (58‐75)*	62 (52‐69)	<.001
Female (%)	4 (28.6)*	190 (57.6)	163 (46.6)*	41 (46.1)	39 (41.9)*	26 (56.5)	.008
Comorbidity							
Hypertension (%)	5 (35.7)	77 (23.3)	118 (33.7)*	39 (43.8)*	40 (43.0) *	21 (45.7)*	<.001
Coronary artery disease (%)	2 (14.3)	19 (5.8)	31 (8.9)	7 (7.9)	9 (9.7)	2 (4.3)	.39
COPD (%)	0 (0)	3 (0.9)	6 (1.7)	2 (2.2)	0 (0)	0 (0)	.613
Malignancy (%)	3 (21.4)*	5 (1.5)	7 (2.0)	0 (0)	2 (2.2)	0 (0)	.009
Chronic kidney disease (%)	1 (7.1)	4 (1.2)	2 (0.6)	1 (1.1)	1 (1.1)	0 (0)	.269
Cerebrovascular disease (%)	2 (14.3)*	8 (2.4)	19 (5.4)*	6 (6.7)*	5 (5.4)	0 (0)	.037
Temperature (°C)	36.4 (36.1‐36.5)	36.5 (36.3‐37.0)	36.6 (36.3‐37.1)	36.6 (36.3‐37.3)	36.6 (36.2‐37.0)	36.5 (36.2‐37.0)	.144
Respiratory (/min)	20.0 (17.5‐21.0)	20.0 (20.0‐21.0)	20.0 (20.0‐23.0)	20.5 (20.0‐26.3)*	22.0 (20.0‐25.0)*	20.0 (20.0‐23.5)	<.001
Pulse (/min)	92.0 (75.0‐99.5)	90.0 (80.0‐100.0)	90.0 (80.0‐102.0)	92.5 (81.3‐108.0)	88.5 (80.0‐102.0)	90.0 (80.0‐107.5)	.328
Diastolic blood pressure (mmHg)	71.0 (66.5‐85.0)	79.5 (71.0‐89.0)	80.0 (72.0‐90.0)	84.0 (74.0‐94.0)	81.5 (74.0‐89.0)	79.5 (67.0‐89.3)	.223
Systolic blood pressure (mmHg)	120.0 (108.5‐142.5)	125.5 (116.0‐141.0)	130.0 (117.0‐144.0)	132.0 (122.0‐149.0)	136.0 (125.0‐147.0)*	133.0 (117.5‐144.5)	.002
Body mass index (BMI)	22.7 (16.5‐24.7)	23.5 (21.1‐25.3)	23.6 (21.5‐25.4)	24.2 (21.9‐26.0)	23.9 (21.6‐25.7)	24.4 (21.9‐25.8)	.402
Laboratory findings							
White blood cell (*10^9/L)	7.0 (4.8‐11.3)	5.6 (4.3‐7.4)	6.1 (4.7‐8.1)*	6.8 (5.0‐8.6)*	6.5 (5.1‐8.6)	6.2 (4.8‐7.7)	<.001
Red blood cell (*10^12/L)	3.1 (2.5‐3.8)*	4.1 (3.7‐4.5)	4.1 (3.7‐4.5)	4.2 (3.8‐4.6)	4.2 (3.8‐4.6)	4.3 (3.9‐4.9)	<.001
Neutrophil (*10^9/L)	4.8 (3.4‐10.1)	3.5 (2.5‐5.0)	4.4 (3.0‐6.2)*	4.7 (3.4‐7.2)*	4.4 (3.2‐6.2)*	4.1 (2.6‐6.1)	<.001
Haemoglobin (g/L)	97.0 (78.3‐124.5)	124.0 (109.8‐135.3)	126.0 (115.5‐137.0)	127.0 (116.5‐138.0)	129.0 (118.0‐141.5)*	128.5 (117.8‐143.0)	<.001
Platelet (*10^9/L)	179.0 (120.0‐274.8)	211.0 (160.0‐262.8)	218.5 (166.0‐293.5)	219.0 (158.5‐293.0)	226.0 (177.0‐299.0)	223.5 (184.0‐281.3)	.224
Alanine transaminase (U/L)	21.5 (13.8‐41.8)	18.0 (12.0‐31.3)	26.0 (17.0‐41.0)*	28.0 (17.5‐41.5)*	24.0 (16.5‐34.5)	17.5 (12.0‐26.5)	<.001
Aspartate transaminase (U/L)	27.0 (19.8‐49.0)	22.0 (17.0‐31.0)	27.0 (20.0‐43.3)*	27.0 (18.5‐42.0)	24.0 (19.0‐33.0)	19.0 (14.0‐28.5)	<.001
Total bilirubin (umol/L)	14.4 (9.6‐33.3)*	8.0 (5.8‐11.2)	9.2 (6.6‐13.2)*	9.0 (6.2‐13.0)	9.9 (6.9‐14.0)*	9.8 (6.6‐12.4)	<.001
Albumin (g/L)	34.2 (27.7‐36.8)*	39.0 (34.7‐42.6)	34.6 (30.7‐38.1)*	33.8 (30.3‐38.2)*	33.8 (31.3‐38.6)*	35.5 (30.6‐40.8)*	<.001
Globulin (g/L)	33.6 (24.6‐37.7)	30.3 (27.3‐33.1)	33.2 (30.0‐36.7)*	34.2 (31.2‐38.1)*	33.4 (30.4‐38.1)*	32.4 (29.2‐35.3)	<.001
Creatinine (mmol/L)	64.0 (46.3‐123.0)	66.0 (55.0‐78.0)	69.0 (58.0‐86.0)	70.0 (56.0‐91.0)	70.0 (55.0‐91.5)	58.0 (50.5‐73.5)	.004
Blood urea nitrogen (mmol/L)	7.9 (5.0‐11.5)*	4.2 (3.3‐5.4)	4.7 (3.5‐6.4)	4.9 (3.8‐7.1)*	5.6 (3.5‐7.5)*	5.0 (3.9‐7.5)	<.001
Uric acid (umol/L)	229.3 (184.0‐300.9)	269.1 (210.5‐333.9)	245.5 (189.0‐318.1)	257.0 (170.4‐349.8)	258.0 (186.7‐328.3)	251.5 (176.5‐299.1)	.080
Total cholesterol (mmol/L)	3.1 (2.4‐4.2)	3.9 (3.3‐4.7)	3.7 (3.2‐4.3)*	3.6 (2.9‐4.2)	4.1 (3.4‐4.9)	4.3 (3.7‐5.2)	<.001
Total triglycerides (mmol/L)	1.1 (1.0‐2.2)	1.2 (0.9‐1.8)	1.3 (1.0‐1.9)	1.5 (1.1‐2.3)*	1.6 (1.2‐2.2)*	1.8 (1.3‐2.3)*	<.001
High‐density lipoprotein cholesterol (mmol/L)	0.6 (0.5‐0.8)*	1.1 (0.9‐1.3)	0.9 (0.8‐1.1)*	0.9 (0.7‐1.0)*	0.8 (0.7‐1.0)*	1.0 (0.8‐1.2)	<.001
Low‐density lipoprotein cholesterol (mmol/L)	2.0 (1.6‐2.7)	2.5 (1.9‐3.1)	2.4 (1.9‐2.9)	2.2 (1.6‐2.7)	2.8 (2.2‐3.6)	2.7 (2.1‐3.5)	<.001
K+ (mmol/L)	4.3 (4.2‐4.8)	4.2 (3.9‐4.4)	4.1 (3.7‐4.5)	4.2 (3.8‐4.7)	4.4 (4.1‐4.7)*	4.3 (4.0‐4.7)	<.001
Lactate dehydrogenase (U/L)	277.0 (181.3‐400.0)	216.0 (180.0‐276.0)	284.0 (215.3‐392.0)*	305.0 (221.5‐432.5)*	278.0 (213.5‐388.0)*	238.5 (183.0‐301.3)	<.001
Prothrombin time (s)	15.3 (14.2‐16.7)*	13.6 (13.1‐14.1)	14.0 (13.4‐14.7)*	13.9 (13.4‐14.9)*	13.7 (13.1‐14.4)*	13.8 (13.0‐14.5)	<.001
Activated partial thromboplastin time (s)	43.4 (39.2‐50.0)*	38.9 (36.1‐42.4)	38.9 (36.0‐43.0)	38.8 (36.8‐43.0)	39.2 (36.1‐43.1)	36.7 (34.7‐40.0)	.006
D‐Dimer (ug/ml)	1.8 (0.6‐3.5)*	0.5 (0.3‐1.2)	1.0 (0.4‐2.2)*	1.2 (0.6‐3.4)*	1.0 (0.5‐2.5)*	0.7 (0.3‐1.9)	<.001
Interleukin 6 (pg/ml)	13.7 (4.4‐37.8)	6.7 (2.8‐24.3)	14.4 (4.2‐44.4)*	10.4 (3.2‐48.1)	10.3 (3.9‐28.7)	9.5 (3.5‐24.7)	.003
Interleukin 8 (pg/ml)	17.1 (9.6‐24.0)	12.8 (7.9‐23.0)	16.8 (9.7‐28.4)	14.6 (8.1‐26.9)	14.1 (8.8‐28.2)	11.4 (7.6‐13.9)	.034
Tumour necrosis factor‐α (pg/ml)	9.3 (5.7‐16.6)	7.8 (6.2‐10.2)	8.6 (6.7‐11.6)	8.6 (6.8‐11.4)	9.4 (7.2‐13.3)*	8.4 (6.2‐10.3)	.016
Interleukin‐1β (pg/ml)	15.9 (6.1‐41.3)	9.2 (6.6‐13.8)	7.7 (6.1‐11.8)	8.3 (6.3‐14.3)	7.4 (5.9‐14.1)	8.0 (6.3‐9.3)	.625
High‐sensitive C reaction protein (pg/ml)	27.4 (7.6‐79.1)	4.6 (1.0‐35.0)	24.9 (5.0‐76.3)*	38.1 (8.0‐98.6)*	36.9 (3.9‐94.8)*	8.3 (1.8‐63.1)	<.001
Erythrocyte sedimentation rate (mm/h)	77.0 (32.8‐103.3)*	18.0 (8.0‐36.5)	37.0 (18.0‐60.0)*	44.0 (19.0‐66.0)*	34.0 (17.5‐65.0)*	30.0 (20.5‐73.3)	<.001
Myoglobin (ug/L)	63.5 (27.5‐135.3)	34.4 (24.9‐54.4)	46.0 (29.4‐87.8)*	45.6 (29.2‐105.9)	46.0 (27.2‐112.5)	35.8 (20.7‐67.8)	<.001
Creatine kinase (U/L)	42.0 (26.5‐192.0)	66.0 (44.0‐98.0)	68.5 (43.0‐131.8)	68.5 (39.5‐130.0)	64.0 (36.3‐118.0)	47.0 (32.0‐85.5)	.101
Creatine kinase‐MB (U/L)	0.7 (0.4‐3.2)	0.7 (0.4‐1.1)	0.7 (0.5‐1.6)	0.8 (0.5‐1.3)	0.7 (0.5‐1.2)	0.8 (0.4‐1.2)	.323
Glucose (mmol/L)	6.7 (5.5‐9.6)	5.3 (4.8‐6.5)	6.4 (5.6‐7.8)*	8.5 (6.8‐11.6)*	11.3 (8.3‐16.6)*	16.2 (12.2‐19.7)*	<.001
Treatments							
Metformin (%)	0 (0)	3 (0.9)	22 (6.3)*	24 (27.0)*	40 (43.0)*	25 (54.3)*	<.001
Insulin (%)	0 (0)	1 (0.3)	6 (1.7)	23 (25.8)*	51 (54.8)*	26 (56.5)*	<.001
SU/GLN (%)	0 (0)	2 (0.6)	8 (2.3)	8 (9.0)*	26 (28.0)*	12 (26.1)*	<.001
A‐GI (%)	0 (0)	5 (1.5)	33 (9.4)*	35 (39.3)*	48 (51.6)*	30 (65.2)*	<.001
Pioglitazone (%)	1 (7.1)*	0 (0)	1 (0.3)	4 (4.5)*	4 (4.3)*	1 (2.2)*	<.001
DPP‐4I (%)	0 (0)	0 (0)	5 (1.4)*	3 (3.4)*	11 (12.0)*	4 (8.7)*	<.001
SGLT‐2I (%)	0 (0)	0 (0)	2 (0.6)	0 (0)	1 (1.1)	0 (0)	.438
Glucocorticoid (%)	4 (28.6)	109 (33.0)	145 (41.4)*	47 (52.8)*	35 (37.6)	18 (39.1)	.018
Oxygen therapy (%)	14 (100.0)*	213 (64.5)	268 (76.6)*	71 (79.8)*	75 (80.6)*	36 (78.3)	<.001
Ventilator (%)	7 (50.0)*	29 (8.8)	63 (18.0)*	24 (27.0)*	18 (19.4)*	5 (10.9)	<.001
Intubate (%)	5 (35.7)*	10 (3.0)	24 (6.9)*	9 (10.1)*	3 (3.2)	3 (6.5)	.001
Mortality (%)	3 (21.4)*	17 (5.2)	40 (11.4)*	17 (19.1)*	14 (15.1)*	6 (13.0)*	<.001

Abbreviations: A‐GI, Alpha‐glycosidase inhibitors; COPD, chronic obstructive pulmonary disease; DPP‐4I, Dipeptidyl peptidase‐4 inhibitors; GLN/SU, Sulphonylureas/glinides; K+, potassium; SGLT‐2I, Sodium‐glucose cotransporter‐2 inhibitors.*P < .05 vs HbA1c among 5%‐5.9%.

Interestingly, we noted that in the subgroup when HbAlc was the lowest (3%‐4.9%), FBG was unexpectedly higher (6.7 mmol/L) than the group with HbAlc levels among 5%‐5.9% (FBG 5.3 mmol/L). Therefore, the high FBG levels in patients with lower HbAlc levels might be partly explained by overcompensated effects for unnoticed hypoglycaemia, similar as the Somogyi effect (See further in discussion).

These data suggested that lower levels of HbAlc (3%‐4.9%) and higher levels of HbAlc (≥6%) were both associated with increased all‐cause mortality of COVID‐19 patients, which required attention in clinical practice.

### 3.2 Association of HbAlc levels with other clinical parameters in COVID‐19 patients

We then performed correlational analyses between HbAlc levels and other clinical parameters (blood routine, hepatic and renal functions, coagulation function, etc) in COVID‐19 patients to shed light on the internal associations between this metabolic indicator and other systemic biochemical indexes. We noted that HbAlc levels were highly correlated (*P* <.0001) with haemoglobin (Hb) and total cholesterol (TC) (Figure S1A‐B), moderately correlated (.0001 < *P*<.001) with albumin (ALB) and highly sensitive C reaction protein (hs‐CRP) (Figure S1C‐D), and relative lowly correlated (.001 < *P*<.01) with low‐density lipoprotein cholesterol (LDL‐C) (Figure S1E). In contrast, we found high correlations (*P* <.0001) of FBG levels with white blood cell (WBC), ALB, urea, high‐density lipoprotein cholesterol (HDL‐C), prothrombin time (PT), D‐dimer, hs‐CRP and myoglobin (MB) (Figure S1F‐M), moderate correlations (.0001 < *P*<.001) with tumour necrosis factor‐α (TNF‐α) and creatine kinase‐MB (CK‐MB) (Figure S1N‐O), and relative low correlations (.001 < *P*<.01) with total triglycerides (TG).

Taken together, HbAlc levels and FBG were both positively correlated with inflammatory biomarker hs‐CRP and negatively correlated with ALB (indicator of liver function). Respectively, HbAlc levels seemed to be specifically correlated with Hb and cholesterol (TC and LDL‐C) levels while FBG was specifically correlated with WBC, urea (kidney function marker), PT and D‐dimer (coagulation function markers), MB and CK‐MB (muscle damage markers), TG and HDL‐C.

These data clearly indicated the differences in HbAlc‐ and FBG‐related biochemical parameters. These associated cofactors might together contribute to the clinical outcome of COVID‐19 patients.

### 3.3 Increased mortality in COVID‐19 patients with newly identified DM

Among these 922 confirmed COVID‐19 patients, 182 were admitted to hospital with a T2DM history. However, we noted an unignorable number of non‐diagnosed patients with high levels of FBG or HbAlc. To investigate whether early diagnosis and treatment influenced the outcome of COVID‐19. We divided these patients into three groups: (a) non‐DM history patients with normal HbAlc levels; (b) DM history patients; and (c) non‐DM history patients with HbAlc ≥ 6.5%, a diagnostic criterion for diabetes according to the American Diabetes Association. Considering that oral glucose tolerance test (OGTT) test was unlikely to be performed under this specific pandemic condition, HbAlc ≥ 6.5% was selected as a diagnostic criterion for identifying previously undiagnosed DM patients. Accordingly, among these 922 enrolled COVID‐19 patients, 533 patients were non‐DM history patients with all‐cause mortality of 7.2%. In contrast, increased mortality (10.4%) was observed in 182 patients with DM history. Interestingly, the mortality was even higher (20.3%) in 187 newly diagnosed DM patients compared with pre‐diagnosed DM patients (Table 3). Notably, the FBG and HbAlc levels were not further up‐regulated (in fact a little lower) in newly diagnosed DM patients compared with pre‐diagnosed DM patients (Table 3), indicating that increased mortality in newly diagnosed DM patients might not be due to increased glucose levels. In the same way, decreased mortality in pre‐diagnosed DM patients compared with newly diagnosed DM patients might not be directly attributed to the levels of FBG or HbAlc. Alternatively, the different outcome in pre‐diagnosed DM and newly diagnosed DM might be due to early treatment in pre‐diagnosed DM patients (See further in discussion).

**TABLE 3:** The characteristics and clinical outcome of COVID‐19 patients with DM

	Patients without DM (n = 553)	Patients with History of DM (n = 182)	Patients with Newly Identified DM (n = 187)	*P* value
Demographics and clinical characteristics				
Age (y)	62 (48‐70)	66 (59‐72)*	66 (56‐73)*	<.001
Female (%)	294 (53.2)	84 (46.2)	85 (45.5)	.09
Comorbidity				
Hypertension (%)	147 (26.6)	101 (55.5)*	52 (27.8)^#^	<.001
Coronary artery disease (%)	35 (6.3)	27 (14.8)*	8 (4.3)^#^	<.001
COPD (%)	8 (1.4)	2 (1.1)	1 (0.5)	.604
Malignancy (%)	12 (2.2)	3 (1.6)	2 (1.1)	.612
Chronic kidney disease (%)	6 (1.1)	3 (1.6)	0 (0)	.251
Cerebrovascular disease (%)	19 (3.4)	11 (6.0)	10 (5.3)	.244
Temperature (°C)	36.6 (36.3‐37.0)	36.5 (36.2‐36.9)	36.7 (36.3‐37.4)^#^	.034
Respiratory (/min)	20 (20‐22)	20 (20‐25)*	20 (20‐25)*	<.001
Pulse (/min)	90 (80‐100)	90 (80‐106)	92 (82‐106)	.082
Diastolic blood pressure (mmHg)	79.0 (71.0‐89.0)	80.0 (70.0‐90.0)	81.5 (73.0‐91.0)	.369
Systolic blood pressure (mmHg)	126.0 (116.0‐142.0)	135.0 (122.0‐147.0)*	132.5 (120.0‐145.0)*	<.001
Body mass index (BMI)	23.4 (21.1‐25.4)	23.7 (22.0‐25.4)	24.8 (22.0‐26.1)*	.029
Laboratory findings				
White blood cell (*10^9/L)	5.7 (4.4‐7.5)	6.3 (4.8‐7.9)*	6.8 (5.1‐8.9)*	<.001
Red blood cell (*10^12/L)	4.1 (3.7‐4.5)	4.1 (3.7‐4.5)	4.2 (3.8‐4.6)	.104
Neutrophil (*10^9/L)	3.8 (2.7‐5.4)	4.3 (3.1‐6.0)*	4.9 (3.2‐7.2)*	<.001
Haemoglobin (g/L)	125.0 (113.0‐136.0)	128.0 (115.0‐138.3)	127.0 (117.0‐141.0)*	.020
Platelet (*10^9/L)	216.5 (164.3‐272.8)	220.0 (165.8‐292.5)	217.0 (161.0‐295.0)	.668
Alanine transaminase (U/L)	21.0 (13.0‐36.5)	22.0 (13.0‐33.0)	27.0 (17.0‐43.0)*^,#^	<.001
Aspartate transaminase (U/L)	24.0 (18.0‐36.0)	23.0 (18.0‐33.0)	28.0 (19.0‐43.0)*^,#^	.005
Total bilirubin (umol/L)	8.5 (6.2‐12.2)	9.1 (6.6‐13.7)	9.3 (6.6‐13.6)	.037
Albumin (g/L)	37.1 (32.6‐40.9)	34.2 (31.3‐39.3)*	34.2 (30.7‐38.4)*	<.001
Globulin (g/L)	31.5 (28.4‐34.7)	32.3 (28.7‐36.1)	34.3 (31.4‐37.9)*^,#^	<.001
Creatinine (mmol/L)	67.0 (55.0‐82.0)	66.0 (54.0‐86.0)	72.0 (58.0‐91.0)*	.030
Blood urea nitrogen (mmol/L)	4.3 (3.3‐5.8)	4.9 (3.8‐6.7)*	5.1 (3.6‐7.7)*	<.001
Uric acid (umol/L)	258.2 (198.0‐326.5)	251.9 (186.6‐322.0)	255.0 (184.2‐340.0)	.444
Total cholesterol (mmol/L)	3.8 (3.2‐4.5)	3.7 (3.2‐4.6)	4.0 (3.3‐4.5)	.289
Total triglycerides (mmol/L)	1.2 (0.9‐1.8)	1.5 (1.1‐2.1)*	1.6 (1.2‐2.2)*	<.001
High‐density lipoprotein cholesterol (mmol/L)	1.0 (0.8‐1.2)	0.9 (0.7‐1.0)*	0.9 (0.8‐1.0)*	<.001
Low‐density lipoprotein cholesterol (mmol/L)	2.4 (1.9‐3.0)	2.4 (1.9‐3.3)	2.4 (1.9‐3.1)	.657
K+ (mmol/L)	4.2 (3.8‐4.4)	4.3 (3.8‐4.7)*	4.3 (3.9‐4.6)*	.002
Lactate dehydrogenase (U/L)	236.5 (192.0‐320.8)	262.5 (207.5‐347.0)*	298.0 (221.8‐436.5)*^,#^	<.001
Prothrombin time (s)	13.7 (13.2‐14.3)	14.0 (13.3‐14.6)	14.0 (13.3‐15.0)*	.002
Activated partial thromboplastin time (s)	39.0 (36.2‐42.6)	38.9 (36.2‐42.9)	38.5 (35.1‐42.7)	.468
D‐Dimer (ug/ml)	0.6 (0.3‐1.7)	0.9 (0.5‐2.1)*	1.1 (0.5‐2.8)*	<.001
Interleukin 6 (pg/ml)	9.1 (3.1‐31.9)	9.8 (3.6‐27.8)	14.0 (4.2‐57.4)	.070
Interleukin 8 (pg/ml)	14.0 (8.3‐24.3)	13.4 (8.0‐27.8)	15.8 (9.3‐28.3)	.133
Tumour necrosis factor‐α (pg/ml)	8.2 (6.4‐10.4)	8.8 (6.5‐11.2)	9.1 (6.7‐13.3)*	.016
Interleukin‐1β (pg/ml)	8.5 (6.5‐12.6)	7.7 (6.0‐9.6)	8.9 (6.3‐15.8)	.244
High‐sensitive C reaction protein (pg/ml)	10.8 (1.7‐50.5)	13.5 (3.2‐67.5)*	37.4 (5.5‐99.9)*^,#^	<.001
Erythrocyte sedimentation rate (mm/h)	25.0 (10.0‐52.5)	33.5 (16.3‐71.5)	37.0 (20.0‐56.0)*	.008
Myoglobin (ug/L)	37.7 (26.2‐63.2)	46.0 (27.2‐85.5)	46.9 (29.7‐122.1)*	.008
Creatine kinase (U/L)	64.0 (42.5‐103.5)	58.5 (34.0‐103.8)	76.0 (45.0‐136.0)^#^	.013
Creatine kinase‐MB (U/L)	0.7 (0.4‐1.2)	0.7 (0.5‐1.2)	0.9 (0.5‐1.7)*	.023
HbA1c (%)	5.8 (5.6‐6.1)	7.8 (6.7‐9.0)*	7.1 (6.6‐8.2)*	<.001
Glucose (mmol/L)	5.7 (5.0‐7.0)	8.9 (6.5‐14.5)*	8.4 (6.4‐12.4)*	<.001
Treatments				
Metformin (%)	7 (1.3)	73 (40.1)*	34 (18.2)*^,#^	<.001
Insulin (%)	0 (0)	76 (41.8)*	31 (16.6)*^,#^	<.001
SU/GLN (%)	1 (0.2)	43 (23.6)*	12 (6.4)*^,#^	<.001
A‐GI (%)	11 (2.0)	88 (48.4)*	52 (27.8)*^,#^	<.001
Pioglitazone (%)	0 (0)	9 (4.9)*	2 (1.1)*^,#^	<.001
DPP‐4I (%)	2 (0.4)	16 (8.8)*	5 (2.7)*^,#^	<.001
SGLT‐2I (%)	0 (0)	3 (1.6)*	0 (0)	.008
Glucocorticoid (%)	203 (36.7)	67 (36.8)	88 (47.1)*^,#^	.035
Oxygen therapy (%)	386 (69.8)	142 (78.0)*	149 (79.7)*	.009
Ventilator (%)	71 (12.8)	31 (17.0)	44 (23.5)*	.002
Intubate (%)	25 (4.5)	11 (6.0)	18 (9.6)*	.037
Mortality (%)	40 (7.2)	19 (10.4)	38 (20.3)*^,#^	<.001

Abbreviations: A‐GI, Alpha‐glycosidase inhibitors; COPD, chronic obstructive pulmonary disease; DPP‐4I, Dipeptidyl peptidase‐4 inhibitors; GLN/SU, Sulphonylureas/glinides; K+, potassium; SGLT‐2I, Sodium‐glucose cotransporter‐2 inhibitors.*P < .05 vs patients without DM, ^#^ *P* < .05 vs patients with history of DM.

### 3.4 Mortality in DM patients treated with different anti‐hyperglycaemic drugs

To evaluate the influence of long‐term anti‐hyperglycaemic drugs (not the short‐term in‐hospital treatment), we then assessed the in‐hospital mortality of COVID‐19 patients with DM history. Most of these patients were treated with anti‐hyperglycaemic drugs regularly before the COVID‐19 pandemic. We noted that the mortality in pre‐diagnosed diabetic COVID‐19 patients without any anti‐hyperglycaemic drugs was 32.4% while the mortality was 7.9% in insulin‐, 4.1% in metformin‐, 0% in SU/GLN‐ and 2.3% in AGI‐treated patients, respectively (Table 4). Because of the limited number of patients, we were unable to further divide patients into single anti‐diabetic drug groups. Nevertheless, these data still suggested that early identification of diabetes and initiation of appropriate treatment might prevent worse outcome of COVID‐19. Furthermore, we compared the difference between insulin therapy alone, insulin plus oral hypoglycaemic drug therapy and oral hypoglycaemic drug therapy alone in the Table S1. However, there was no difference of mortality between insulin along treated patients and patients without hypoglycaemic drug. Besides, the mortality in the oral hypoglycaemic group was much lower than that in the insulin group (1.4% vs 21.4%, *P* =.014). These results indicated that insulin therapy alone did not reduce mortality or even increased the risk of death in COVID‐19 patients with diabetes.

**TABLE 4:** The characteristics and clinical outcome of COVID‐19 patients with DM treated with different anti‐hyperglycaemic drugs

	No drugs (n = 37)	Insulin (n = 76)	Metformin (n = 73)	SU/GLN (n = 43)	AGI (n = 88)	*P* value
Demographics and clinical characteristics						
Age (y)	69 (61‐79)	66 (61‐72)	62 (55‐70)	67 (60‐73)	66 (57‐72)	.050
Female (%)	16 (43.2)	40 (52.6)	35 (47.9)	19 (44.2)	43 (48.9)	.869
Comorbidity						
Hypertension (%)	24 (64.9)	44 (57.9)	38 (52.1)	21 (48.8)	51 (58.0)	.595
Coronary artery disease (%)	12 (32.4)	8 (10.5)*	3 (4.1)*	6 (14.0)*	10 (11.4)*	.002
COPD (%)	1 (2.7)	1 (1.3)	0 (0)	0 (0)	1 (1.1)	.644
Malignancy (%)	2 (5.4)	1 (1.3)	0 (0)*	0 (0)	1 (1.1)	.173
Chronic kidney disease (%)	1 (2.7)	2 (2.6)	0 (0)	0 (0)	1 (1.1)	.505
Cerebrovascular disease (%)	3 (8.1)	5 (6.6)	2 (2.7)	2 (4.7)	5 (5.7)	.718
Temperature (°C)	36.6 (36.4‐36.9)	36.5 (36.2‐36.9)	36.5 (36.2‐37.0)	36.3 (36.1‐36.7)	36.5 (36.2‐36.8)	.140
Respiratory (/min)	20 (20‐28)	22 (20‐25)	21 (20‐25)	21 (19‐24)	21 (20‐25)	.863
Pulse (/min)	93 (80‐108)	90 (80‐104)	93 (80‐107)	90 (81‐102)	90 (80‐101)	.876
Diastolic blood pressure (mmHg)	78.0 (70.0‐88.0)	80.0 (70.0‐89.5)	80.0 (70.0‐88.0)	80.0 (70.5‐89.5)	81.0 (74.0‐90.0)	.712
Systolic blood pressure (mmHg)	126.0 (120.0‐147.0)	137.0 (125.0‐151.0)	133.0 (120.0‐142.0)	136.0 (126.0‐147.5)	136.0 (125.0‐147.0)	.249
Laboratory findings						
White blood cell (*10^9/L)	6.8 (5.2‐10.2)	5.8 (4.7‐7.6)	5.9 (4.6‐7.4)	6.1 (5.2‐7.9)	5.8 (4.6‐7.7)	.242
Red blood cell (*10^12/L)	3.9 (3.5‐4.3)	4.1 (3.7‐4.6)	4.2 (3.8‐4.7)	4.1 (3.9‐4.5)	4.1 (3.7‐4.4)	.071
Neutrophil (*10^9/L)	5.3 (3.4‐8.4)	4.3 (3.0‐6.0)	4.0 (2.9‐5.4)	4.2 (3.5‐5.6)	4.0 (3.0‐5.6)	.078
Haemoglobin (g/L)	123.0 (102.5‐130.5)	128.0 (115.3‐138.0)	129.0 (117.0‐140.0)	127.0 (119.0‐139.0)	128.0 (116.5‐138.0)	.203
Platelet (*10^9/L)	201.0 (146.5‐288.5)	223.0 (178.0‐297.0)	231.0 (177.5‐321.0)	223.0 (189.0‐300.0)	215.0 (166.3‐264.3)	.380
Alanine transaminase (U/L)	20.0 (12.5‐34.5)	22.0 (12.0‐34.8)	21.0 (13.0‐34.5)	22.0 (16.0‐29.0)	21.0 (15.0‐31.8)	.995
Aspartate transaminase (U/L)	30.0 (19.0‐47.0)	21.0 (17.3‐31.0)	21.0 (16.0‐31.0)*	21.0 (17.0‐30.0)	22.0 (17.0‐31.0)	.034
Total bilirubin (umol/L)	9.9 (6.2‐15.1)	9.7 (6.6‐14.5)	8.3 (6.4‐11.5)	9.3 (6.6‐13.5)	8.5 (6.4‐12.5)	.550
Albumin (g/L)	34.3 (30.9‐36.2)	33.4 (30.5‐38.3)	34.9 (30.7‐40.7)	34.9 (31.9‐40.5)	34.1 (30.5‐39.7)	.187
Globulin (g/L)	33.8 (27.8‐36.3)	33.5 (30.1‐36.6)	32.1 (28.8‐36.8)	31.1 (28.6‐34.6)	32.3 (28.8‐36.1)	.555
Creatinine (mmol/L)	66.0 (52.5‐97.5)	62.5 (52.0‐87.0)	66.0 (56.0‐78.0)	70.0 (55.0‐84.0)	64.0 (54.3‐81.0)	.754
Blood urea nitrogen (mmol/L)	5.5 (3.8‐9.2)	4.9 (3.6‐7.3)	4.7 (3.9‐5.9)	4.6 (3.9‐6.1)	4.6 (3.7‐6.1)	.174
Uric acid (umol/L)	259.0 (192.4‐324.0)	232.0 (170.8‐294.1)	256.0 (192.7‐323.9)	235.0 (190.0‐317.0)	224.8 (158.3‐295.5)	.451
Total cholesterol (mmol/L)	3.4 (3.0‐3.8)	4.1 (3.4‐5.0)*	4.0 (3.3‐4.9)*	3.6 (3.2‐5.1)	3.7 (3.2‐4.6)	.009
Total triglycerides (mmol/L)	1.4 (1.1‐2.3)	1.4 (1.0‐2.2)	1.5 (1.1‐2.2)	1.4 (1.0‐1.9)	1.4 (1.0‐1.9)	.793
High‐density lipoprotein cholesterol (mmol/L)	0.8 (0.6‐1.0)	0.9 (0.7‐1.0)	0.9 (0.7‐1.1)	0.8 (0.7‐1.1)	0.9 (0.8‐1.1)	.307
Low‐density lipoprotein cholesterol (mmol/L)	2.2 (1.7‐2.6)	2.8 (2.2‐3.6)*	2.7 (2.1‐3.4)	2.5 (1.9‐3.5)	2.5 (2.1‐3.2)	.035
K+ (mmol/L)	4.3 (4.0‐4.8)	4.3 (3.8‐4.7)	4.2 (3.8‐4.5)	4.4 (4.0‐4.9)	4.3 (3.8‐4.6)	.786
Lactate dehydrogenase (U/L)	309.0 (225.5‐587.0)	273.0 (220.3‐337.0)	247.0 (195.5‐322.5)*	241.0 (202.0‐300.0)*	241.0 (202.3‐300.0)*	.005
Prothrombin time (s)	14.4 (13.5‐15.9)	13.8 (13.3‐14.5)	13.8 (13.1‐14.4)	13.7 (12.9‐14.4)*	13.8 (13.2‐14.5)	.023
Activated partial thromboplastin time (s)	39.9 (36.8‐44.8)	38.3 (35.4‐41.5)	38.4 (36.5‐41.8)	38.8 (36.3‐43.1)	38.4 (36.1‐41.8)	.470
D‐Dimer (ug/ml)	1.6 (0.6‐4.1)	1.0 (0.6‐2.3)	0.8 (0.3‐1.5)*	0.6 (0.4‐1.1)*	0.8 (0.4‐1.8)	.009
Interleukin 6 (pg/ml)	18.5 (8.8‐48.6)	8.4 (3.7‐21.7)	7.6 (3.2‐25.4)	7.4 (3.2‐12.8)*	7.2 (3.6‐20.9)	.031
Interleukin 8 (pg/ml)	21.5 (11.4‐43.7)	12.3 (8.2‐25.1)	11.5 (8.0‐20.9)	10.7 (7.1‐20.8)*	11.0 (7.3‐20.2)*	.021
Tumour necrosis factor‐α (pg/ml)	9.5 (6.9‐12.3)	9.7 (6.9‐11.8)	7.9 (6.3‐11.0)	8.7 (6.4‐10.0)	8.7 (6.3‐10.9)	.127
Interleukin‐1β (pg/ml)	9.2 (6.7‐15.3)	7.4 (5.8‐9.3)	6.8 (5.4‐11.9)	8.9 (5.7‐10.3)	8.1 (6.5‐11.6)	.616
High‐sensitive C reaction protein (pg/ml)	49.9 (5.8‐93.2)	16.1 (3.2‐85.4)	10.8 (2.8‐40.6)	8.0 (2.8‐47.8)	12.7 (2.7‐60.2)	.103
Erythrocyte sedimentation rate (mm/h)	40.0 (11.5‐72.5)	33.0 (19.0‐85.0)	34.0 (18.3‐77.8)	30.0 (15.8‐49.0)	27.0 (16.0‐63.0)	.482
Myoglobin (ug/L)	84.3 (39.2‐193.1)	40.1 (24.0‐78.8)*	38.1 (25.1‐54.7)*	45.9 (27.3‐66.4)	40.0 (25.2‐61.3)*	.009
Creatine kinase (U/L)	88.0 (56.0‐251.0)	42.0 (26.0‐71.5)*	51.0 (31.5‐87.0)	43.0 (30.0‐86.5)*	52.0 (30.5‐81.5)*	.003
Creatine kinase‐MB (U/L)	1.0 (0.7‐3.0)	0.6 (0.4‐1.1)*	0.6 (0.4‐1.0)*	0.7 (0.5‐1.1)	0.6 (0.4‐1.1)*	.013
HbA1c (%)	6.4 (6.1‐7.4)	8.6 (7.9‐10.0)*	8.3 (7.2‐9.9)*	8.5 (7.0‐9.5)*	8.2 (7.0‐9.2)*	<.001
Glucose (mmol/L)	7.3 (5.5‐8.8)	11.4 (8.2‐17.4)*	10.3 (7.0‐15.7)*	10.4 (6.7‐13.6)	10.5 (6.7‐15.7)*	.001
Treatments						
Oxygen therapy (%)	33 (89.2)	61 (80.3)	58 (79.5)	33 (76.7)	66 (75.0)	.494
Ventilator (%)	15 (40.5)	12 (15.8)*	7 (9.6)*	3 (7.0)*	9 (10.2)*	<.001
Intubate (%)	10 (27.0)	0 (0)*	1 (1.4)*	0 (0)*	1 (1.1) b	<.001
Mortality (%)	12 (32.4)	6 (7.9)*	3 (4.1)*	0 (0)*	2 (2.3)*	<.001

Abbreviations: COPD, chronic obstructive pulmonary disease; K+, potassium.*P < .05 vs no anti‐hyperglycaemic drugs.

## 4 DISCUSSION

In this study, we analysed the association of HbAlc levels with clinical outcome of COVID‐19 patients, finding that all‐cause mortality was increased in patients with lower levels of HbAlc (3%‐4.9%) and higher levels of HbAlc (≥6%) compared with HbAlc levels between 5% and 5.9%. These data suggested that HbAlc might be a potential prognostic marker for assessing the risk of death in COVID‐19 patients. Furthermore, by using the criterion of HbAlc ≥ 6.5%, we observed further increase of mortality in patients with newly identified DM compared with pre‐diagnosed DM. Moreover, in patients with pre‐diagnosed DM, the mortality was decreased in patients treated with anti‐hyperglycaemic drugs. Early identification of diabetes and initiation of appropriate treatment might be vital to improve clinical outcome in COVID‐19 patients.

Diabetes has been diagnosed for decades with fasting plasma glucose (FPG) test or with an oral glucose tolerance test (OGTT).^13^ However, it was suggested that a clinical parameter describing the extent of a biological phenomenon over a long period provides a more robust indicator of glycaemia than a parameter describing it in the short term or in a given moment only.^13^, ^14^ The measurement of HbAlc equals the assessment of hundreds of fasting glucose levels as well as postprandial glucose peaks; therefore, it is a more robust and reliable measurement than FPG plasma glucose.^15^ Interestingly, our data showed different observations that lower HbAlc levels (3%‐4.9%) were associated with increased mortality, while lower FBG levels (3‐4.9mmol/l) were associated with decreased mortality. Moreover, FBG levels were increased (6.7mmol/L) in patients with lower levels of HbAlc (3%‐4.9%), suggesting that these patients with high FBG levels might not be really ‘diabetic’ patients. The high FBG levels in patients with lower HbAlc levels might be partly explained by overcompensated effects for unnoticed hypoglycaemia, similar as the Somogyi effect.

The Somogyi effect, also known as the ‘chronic Somogyi rebound’ or ‘post hypoglycemic hyperglycemia’, was described that when blood glucose levels dropped too low during the late evening, activation of counter‐regulatory hormones such as adrenaline, corticosteroids, growth hormone and glucagon might be observed, leading to activation of gluconeogenesis and resultant hyperglycaemia in the early morning.^16^ To further explore the potential relationship, we also divided the group with HbA1c between 3% and 4.9% into two subgroups according to whether the FBG level was higher than 7 mmol/L, one of the diagnostic criteria for diabetes. The mortality in group with FBG higher than 7 mmol/L was much higher than in group with FBG lower than 7 mmol/L (37.5% vs 0%, *P* =.209). Because of the small number of patients (6 vs 8) in the two groups, there was no statistical difference. However, it was still an indicator that COVID‐19 patients with lower HbA1c levels and higher FBG levels had increased risk of death. Studies had shown that a hypoglycaemic episode could put patients at risk for neurological dysfunction, coma or even death. With repeated hypoglycaemia, the counter‐regulatory system that was supposed to keep blood glucose levels in range will start to fail.^17^ Patients with COVID‐19 tend to have multiple organ dysfunction and hypoglycaemia induced increase of adrenaline, corticosteroids, growth hormone and glucagon as well as dysregulated blood glucose homeostasis might together exacerbate the patients’ condition. To test this hypothesis, future study will necessitate more critical blood glucose monitoring on patients co‐exist with lower HbAlc levels and high FBG levels to uncover potential hypoglycaemia in avoid of increased mortality.

To reveal the hidden network between metabolic disorder and multiple organ dysfunction, the association of HbAlc levels and FBG levels with other clinical parameters was evaluated. We noted that HbAlc levels and FBG were both positively correlated with inflammatory biomarker hs‐CRP and negatively correlated with ALB (indicator of liver function). Specifically, HbAlc levels were positively correlated with Hb and cholesterol (TC and LDL‐C) levels while FBG was specifically correlated with WBC, Urea (kidney function marker), PT and D‐dimer (coagulation function markers), MB and CK‐MB (muscle damage markers). These data indicated that hyperglycaemia was closely related to inflammation, hepatic injury, coagulation disorders, etc, which might collectively promote COVID‐19 patients become more serious. HbA1c is the non‐enzymatic glycosylation product of Hb, which could explain the correlation between them. Besides, liver is an important organ for glycogen production and gluconeogenesis, which is of great importance for the regulation of blood glucose homeostasis. Meanwhile, TC and ALB are also synthesized by the liver, reflecting the function of liver. Therefore, it is not difficult to understand the correlation between HbA1c and liver function indicators. Notably, according to animal studies, inflammatory markers such as TNF‐α and IL6 were significantly increased in diabetic mice model,^18^ indicating that diabetes could directly result in accelerating inflammation. So, patients with poor glycaemic control might tend to have a more severe inflammatory status, which could partly explain the correlation between HbA1c and hs‐CRP. However, the cause‐effect relationships between hyperglycaemia and other clinical parameters remain to be determined in the future.

Interestingly, we noted that the mortality of COVID‐19 in newly diagnosed DM patients was higher than pre‐diagnosed DM patients. Unexpectedly, the FBG levels and HbAlc levels were not further increased in newly diagnosed DM patients compared with pre‐diagnosed DM patients. These data suggested that increased mortality in newly diagnosed DM patients might not be directly linked to blood glucose levels. It is possible that the long‐termed anti‐hyperglycaemia treatment in pre‐diagnosed DM patients might have additional effects such as the anti‐inflammatory property. Interestingly, previous studies have demonstrated that anti‐inflammatory properties of metformin are exerted irrespective of diabetes mellitus status.^19^ Other study also showed that patients with newly diagnosed T2DM exhibited a marked chronic inflammatory state characterized by increased IL‐6, TNF‐α, IL‐1β, IL‐2 and ferritin levels. After 1 year of treatment with acarbose or metformin, IL‐6, TNF‐α, IL‐1β and ferritin levels were significantly decreased compared with the baseline. The anti‐inflammatory effects of acarbose and metformin were comparable and required a long‐term treatment (1 year).^20^ While recent study has suggested that host inflammatory response becomes a major cause of lung damage and subsequent mortality during COVID‐19,^21^ its entirely possible that anti‐hyperglycaemic drugs such as acarbose and metformin protected against inflammatory damage caused by COVID‐19 through their potential beyond glucose‐lowering effects. These possibilities are intriguing subjects for future studies.

In terms of the insulin treatment, it should be noted that in this study, only DM patients treated with regularly basal levels of long‐acting or intermediate‐acting insulin analogues were considered as insulin users. Short‐acting human regular insulin was not included. Therefore, the effects of in‐hospital short‐term insulin treatment (or other oral anti‐diabetic drugs) are still unclear and remain to be determined.

The limitations of this study are as follows: (a) The number of COVID‐19 patients with HbAlc level detected was relatively small, which might not represent COVID‐19 patients in general. (b) The onset time (disease courses) of these newly identified DM patients were not clear, which might also influence the outcome of COVID‐19. 3) We only evaluated association of all‐cause mortality with long‐termed anti‐hyperglycaemic therapy in COVID‐19 patients pre‐diagnosed with DM. The short‐term anti‐hyperglycaemic treatment in newly identified DM patients were not included.

In summary, our data investigated the association of HbAlc with mortality in COVID‐19 patients, finding that all‐cause mortality was increased in patients with lower levels of HbAlc (3%‐4.9%) and higher levels of HbAlc (≥6%). Moreover, in patients with pre‐diagnosed DM, the mortality was decreased in patients treated with anti‐hyperglycaemic drugs. These findings suggested that early identification of diabetes and initiation of appropriate treatment might be vital to improve clinical outcome in COVID‐19 patients.

## AUTHOR CONTRIBUTIONS

**shuai yuan:** Formal analysis (equal). **Huaping Li:** Writing‐original draft (equal). **Chen Chen:** Conceptualization (equal); Validation (equal). **Feng Wang:** Data curation (equal); Methodology (equal); Visualization (equal). **Dao Wen Wang:** Project administration (equal).

## Supporting information

Fig S1

Table S1
