# Supplementary figures and images for: Association of glycosylated haemoglobin HbA1c levels with outcome in patients with COVID‐19: A Retrospective Study
Source: J Cell Mol Med. 2021 Mar 10;25(7):3484–97. doi: 10.1111/jcmm.16431 (PMC8034481; doi:10.1111/jcmm.16431)

# Supplement Figure

# Association of HbA1c levels with other clinical parameters

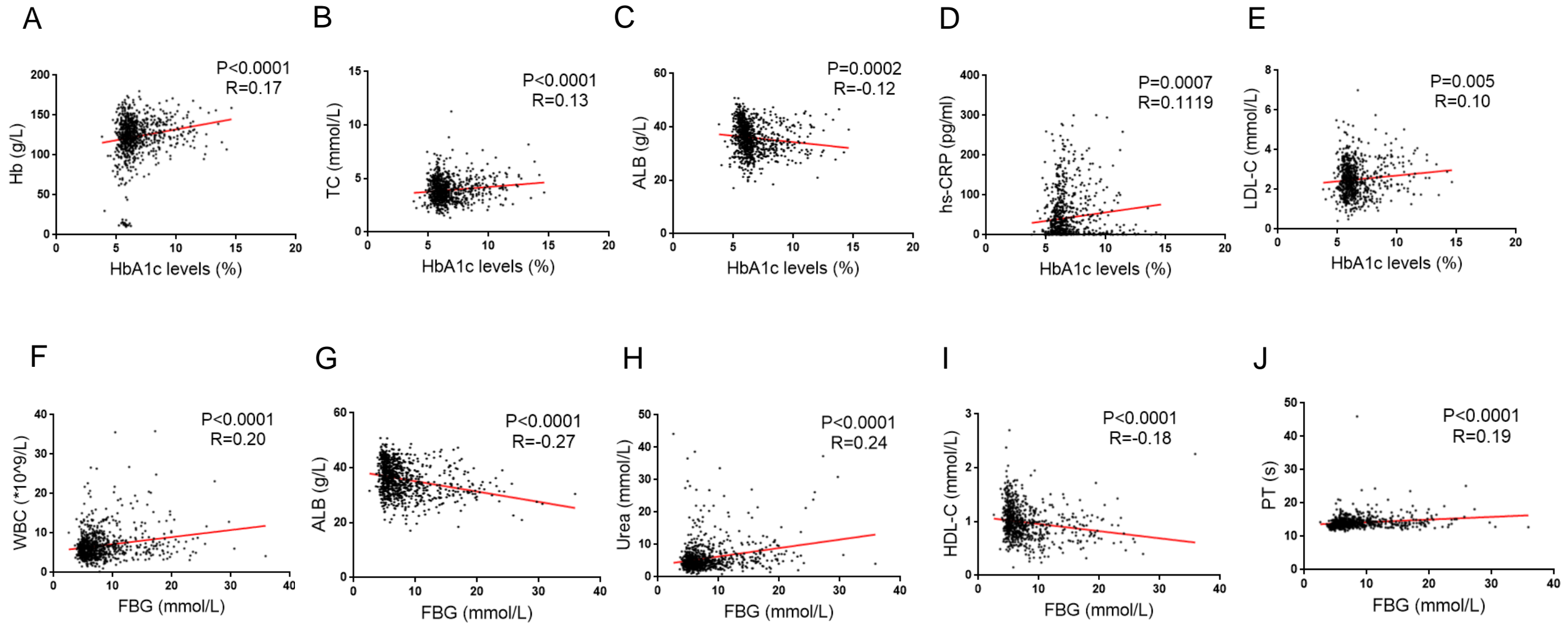

K

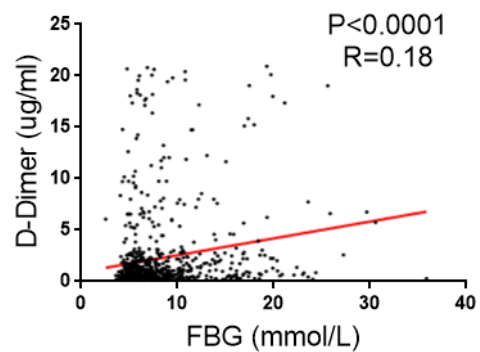

L

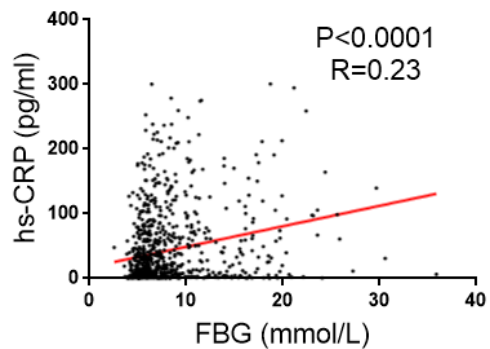

M

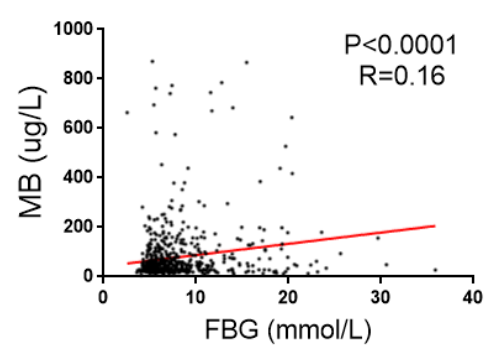

N

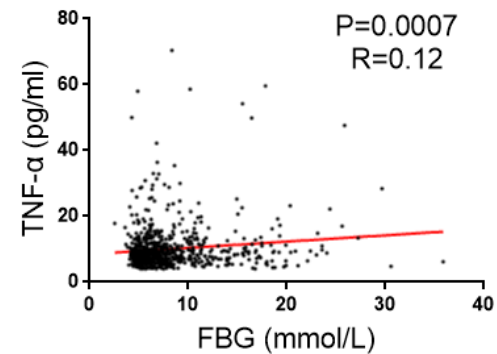

O

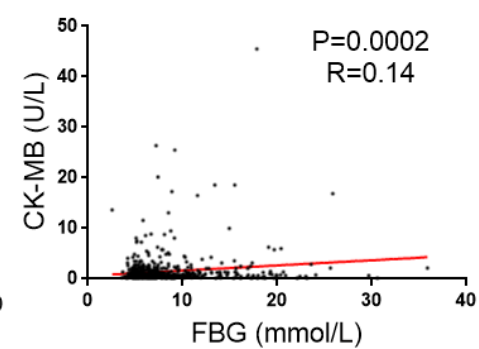

P

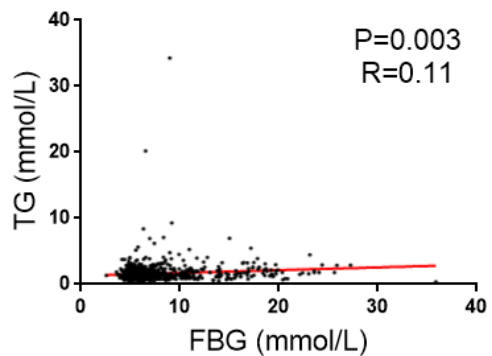

Supplement: Supplementary file 1 — Fig S1 [file JCMM-25-3484-s002.pdf]
