# Supplementary material for: Association of glycosylated haemoglobin HbA1c levels with outcome in patients with COVID‐19: A Retrospective Study
Source: J Cell Mol Med. 2021 Mar 10;25(7):3484–97. doi: 10.1111/jcmm.16431 (PMC8034481; doi:10.1111/jcmm.16431)
Supplement: Supplementary file 2 — Table S1 [file JCMM-25-3484-s001.docx]

**Supplement Table. The characteristics and clinical outcomes of COVID-19 patients with DM treated with insulin or hypoglycemic drugs.**

|  | **No Drugs**  **(n=37)** | **Insulin along**  **(n=14)** | **Insulin plus** **oral hypoglycemic drugs**  **(n=62)** | **Oral hypoglycemic drugs (n=69)** | **P value** |
| --- | --- | --- | --- | --- | --- |
| Age (y) | 69 (62-80) | 66 (60-71) | 67 (61-72) | 65 (56-72) | 0.151 |
| Female (%) | 16 (43.2) | 6 (42.9) | 34 (54.8) | 28 (40.6) | 0.402 |
| Comorbidity |  |  |  |  |  |
| Hypertension (%) | 24 (64.9) | 6 (42.9) | 38 (61.3) | 33 (47.8) | 0.195 |
| Coronary artery disease (%) | 12 (32.4) | 2 (14.3) | 6 (9.7) * | 7 (10.1) * | 0.019 |
| COPD (%) | 1 (2.7) | 0 (0) | 1 (1.6) | 0 (0) | 0.459 |
| Malignancy (%) | 2 (5.4) | 0 (0) | 1 (1.6) | 0 (0) | 0.188 |
| Chronic kidney disease (%) | 1 (2.7) | 1 (7.1) | 1 (1.6) | 0 (0) | 0.267 |
| Cerebrovascular disease (%) | 3 (8.1) | 2 (14.3) | 3 (4.8) | 3 (4.3) | 0.563 |
| Temperature (°C) | 36.6 (36.4-36.9) | 36.5 (36.2-36.9) | 36.5 (36.2-36.9) | 36.5 (36.2-36.8) | 0.281 |
| Respiratory (/min) | 20 (20-28) | 21 (20-25) | 22 (20-25) | 20 (20-23) | 0.476 |
| Pulse (/min) | 93 (80-108) | 99 (78-109) | 90 (80-102) | 90 (80-105) | 0.916 |
| Diastolic blood pressure (mmHg) | 78.0 (70.0-88.0) | 82 (77-87) | 80 (70-90) | 83 (74-91) | 0.479 |
| Systolic blood pressure (mmHg) | 126.0 (120.0-147.0) | 137 (124-156) | 137 (125-149) | 135 (121-146) | 0.519 |
| White blood cell (*10^9/L) | 6.8 (5.2-10.2) | 6.0 (4.8-9.1) | 5.8 (4.7-7.5) | 6.4 (4.9-7.9) | 0.423 |
| Red blood cell (*10^12/L) | 3.9 (3.5-4.3) | 4.1 (3.7-4.5) | 4.1 (3.8-4.6) | 4.2 (3.8-4.8) * | 0.029 |
| Neutrophil (*10^9/L) | 5.3 (3.4-8.4) | 4.3 (2.9-8.5) | 4.2 (3.0-6.0) | 4.2 (3.2-5.5) | 0.168 |
| Hemoglobin (g/L) | 123.0 (102.5-130.5) | 124.5 (102.5-139.3) | 128.0 (117.5-138.0) | 129.0 (119.5-144.0) * | 0.048 |
| Platelet (*10^9/L) | 201.0 (146.5-288.5) | 265.0 (184.3-295.5) | 221.5 (175.8-298.5) | 220.0 (167.5-289.5) | 0.660 |
| Alanine transaminase (U/L) | 20.0 (12.5-34.5) | 23.5 (12.8-41.3) | 21.5 (12.0-33.5) | 23.0 (15.0-31.5) | 0.793 |
| Aspartate transaminase (U/L) | 30.0 (19.0-47.0) | 25.0 (19.8-37.5) | 20.5 (17.0-30.3) * | 24.0 (17.0-31.5) | 0.020 |
| Total bilirubin (umol/L) | 9.9 (6.2-15.1) | 10.8 (7.5-15.7) | 9.2 (6.2-14.8) | 8.5 (7.2-12.0) | 0.579 |
| Albumin (g/L) | 34.3 (30.9-36.2) | 33.3 (31.4-38.4) | 33.4 (30.5-38.3) | 36.9 (33.0-42.4) | 0.009 |
| Globulin (g/L) | 33.8 (27.8-36.3) | 33.7 (30.3-37.9) | 33.4 (30.0-36.4) | 30.7 (27.6-34.4) | 0.148 |
| Creatinine (mmol/L) | 66.0 (52.5-97.5) | 63.5 (48.0-104.8) | 61.5 (52.8-87.0) | 70.0 (57.5-81.0) | 0.684 |
| Blood urea nitrogen (mmol/L) | 5.5 (3.8-9.2) | 5.3 (3.1-11.2) | 4.9 (3.7-7.1) | 4.6 (3.9-6.1) | 0.230 |
| Uric acid (umol/L) | 259.0 (192.4-324.0) | 256.5 (211.1-361.7) | 220.5 (160.8-284.4) | 267.0 (208.5-323.9) | 0.285 |
| Total cholesterol (mmol/L) | 3.4 (3.0-3.8) | 4.3 (3.4-5.3) * | 4.0 (3.2-4.9) * | 3.7 (3.1-4.6) | 0.007 |
| Total triglycerides (mmol/L) | 1.4 (1.1-2.3) | 1.7 (1.2-2.0) | 1.4 (1.0-2.2) | 1.5 (1.1-2.0) | 0.855 |
| High density lipoprotein cholesterol (mmol/L) | 0.8 (0.6-1.0) | 0.9 (0.7-1.0) | 0.9 (0.7-1.1) | 0.9 (0.8-1.1) | 0.436 |
| Low density lipoprotein cholesterol (mmol/L) | 2.2 (1.7-2.6) | 2.6 (1.8-3.6) | 2.9 (2.2-3.5) * | 2.3 (1.9-3.2) | 0.012 |
| K+ (mmol/L) | 4.3 (4.0-4.8) | 4.3 (3.8-4.8) | 4.3 (3.8-4.6) | 4.2 (3.8-4.6) | 0.971 |
| Lactate dehydrogenase (U/L) | 309.0 (225.5-587.0) | 291.5 (222.8-368.8) | 270.0 (219.3-335.0) | 228.0 (193.5-295.5) * | 0.002 |
| Prothrombin time (s) | 14.4 (13.5-15.9) | 14.0 (13.7-14.6) | 13.8 (13.2-14.5) | 13.8 (13.0-14.6) | 0.058 |
| Activated partial thromboplastin time (s) | 39.9 (36.8-44.8) | 38.3 (35.5-41.6) | 38.3 (35.1-41.5) | 38.8 (36.6-43.7) | 0.361 |
| D-Dimer (ug/ml) | 1.6 (0.6-4.1) | 0.9 (0.5-4.0) | 1.0 (0.6-2.1) | 0.6 (0.3-1.3) * | 0.005 |
| Interleukin 6 (pg/ml) | 18.5 (8.8-48.6) | 10.2 (3.2-38.1) | 8.4 (3.9-20.2) | 7.2 (3.1-29.6) | 0.081 |
| Interleukin 8 (pg/ml) | 21.5 (11.4-43.7) | 30.2 (10.5-43.6) | 11.3 (8.0-19.0) * | 12.4 (7.2-24.0) | 0.006 |
| Tumor necrosis factor-α (pg/ml) | 9.5 (6.9-12.3) | 11.0 (7.8-20.0) | 9.3 (6.6-11.5) | 7.7 (5.6-9.6) | 0.011 |
| Interleukin-1β (pg/ml) | 9.2 (6.7-15.3) | 7.5 (5.1-7.5) | 7.3 (5.9-9.8) | 7.7 (5.7-9.0) | 0.518 |
| High-sensitive C reaction protein (pg/ml) | 49.9 (5.8-93.2) | 4.1 (2.6-120.5) | 17.2 (3.7-82.3) | 9.7 (2.8-41.5) | 0.078 |
| Erythrocyte sedimentation rate (mm/h) | 40.0 (11.5-72.5) | 70.0 (16.0-88.0) | 32.0 (19.0-85.0) | 28.5 (15.0-49.0) | 0.302 |
| Myoglobin (ug/L) | 84.3 (39.2-193.1) | 27.7 (20.1-79.3) | 41.7 (24.1-77.6) * | 45.9 (27.2-62.8) * | 0.016 |
| Creatine kinase (U/L) | 88.0 (56.0-251.0) | 60.0 (36.0-109.0) | 34.0 (26.0-69.5) * | 57.0 (40.0-92.0) | <0.001 |
| Creatine kinase-MB (U/L) | 1.0 (0.7-3.0) | 0.5 (0.3-0.8) | 0.6 (0.4-1.2) * | 0.6 (0.4-1.1) * | 0.013 |
| HbA1c (%) | 6.4 (6.1-7.4) | 8.6 (7.5-9.5) * | 8.6 (8.0-10.1) * | 7.3 (6.6-8.6) * | <0.001 |
| Glucose (mmol/L) | 7.3 (5.5-8.8) | 13.3 (7.3-19.3) * | 11.4 (8.3-17.0) * | 8.1 (6.1-13.0) | <0.001 |
| Oxygen therapy (%) | 33 (89.2) | 8 (57.1) * | 53 (85.5) | 48 (69.6) * | 0.011 |
| Ventilator (%) | 15 (40.5) | 1 (7.1) * | 11 (17.7) * | 4 (5.8) * | <0.001 |
| Intubate (%) | 10 (27.0) | 0 (0) * | 0 (0) * | 1 (1.4) * | <0.001 |
| Mortality (%) | 12 (32.4) | 3 (21.4) | 3 (4.8) * | 1 (1.4) * | <0.001 |

* P<0.05 vs. no anti-hyperglycemic drugs
